# Effect of Flow and Particle-Plastron Collision on the Longevity of Superhydrophobicity

**DOI:** 10.1038/srep41448

**Published:** 2017-01-27

**Authors:** Babak Vajdi Hokmabad, Sina Ghaemi

**Affiliations:** 1 Department of Mechanical Engineering, University of Alberta, Edmonton, AB, T6G 1H9, Canada

## Abstract

Among diverse methods for drag reduction, superhydrophobicity has shown considerable promise because it can produce a shear-free boundary without energy input. However, the plastron experiences a limited lifetime due to the dissolution of trapped air from surface cavities, into the surrounding water. The underwater longevity of the plastron, as it is influenced by environmental conditions, such as fine particles suspended in the water, must be studied in order to implement superhydrophobicity in practical applications. We present a proof-of-concept study on the kinetics of air loss from a plastron subjected to a canonical laminar boundary layer at Re_*δ*_ = 1400 and 1800 (based on boundary layer thickness) with and without suspending 2 micron particles with density of 4 Kg/m^3^. To monitor the air loss kinetics, we developed an *in situ* non-invasive optical technique based on total internal reflection at the air-water interface. The shear flow at the wall is characterized by high resolution particle image velocimetry technique. Our results demonstrate that the flow-induced particle-plastron collision shortens the lifetime of the plastron by ~50%. The underlying physics are discussed and a theoretical analysis is conducted to further characterize the mass transfer mechanisms.

Since its discovery, superhydrophobicity has attracted significant attention in terms of both fundamental science^1^,^2^,^3^,^4^ and technological applications^5^,^6^,^7^,^8^,^9^,^10^. Recent research has revealed biomimetic superhydrophobic surfaces in transport phenomena^11^,^12^. As a result, researchers have focused on questions ranging from fundamental non-wetting properties to potential underwater applications as drag reducing^5^,^6^,^8^,^11^,^12^,^13^,^14^ and antibiofouling^15^,^16^ surfaces. The key mechanism behind the functionality of a superhydrophobic surface is acquired through the synergy between micro- or nano-scale surface topography and materials with low surface energy. Such combinations create an air interlayer (a.k.a. plastron) at the interface between the cavities of the solid surface and the liquid phase (Cassie-Baxter state). Consequently, a liquid flow acquires a slip velocity over the surface^3^,^14^,^17^, leading to lower viscous friction compared to a smooth surface. However, in spite of promising drag reduction in laminar^18^,^19^ and turbulent^5^,^11^,^12^ flows, the underwater longevity of plastron hinders the long-term practicality of superhydrophobic surfaces.

Due to its impermanence, superhydrophobicity is not present in underwater environments found in nature^20^. Herminghaus^21^ observed the underwater depletion of the plastron of *Cotinus coggygria Scop* which is a natural superhydrophobic surface. According to Cassie-Baxter (CB) state, the plastron is expected to be thermodynamically stable^2^. However, in reality, the water phase and the air layer are rarely in thermodynamic equilibrium^20^. This non-equilibrium is due to the diffusion of air into water and condensation of water vapor inside the plastron on the non-wetted cavities^22^. The plastron of the bio-inspired synthetic superhydrophobic surfaces dissolves into water and gradually decays^7^,^20^,^21^,^22^,^23^,^24^,^25^ giving the plastron a lifetime which is a function of not just the characteristics of the surface (i.e. morphology and surface energy), but also the properties of the wetting liquid (e.g., surface tension, temperature, hydrostatic pressure^26^ and dissolved air concentration)^7^,^22^.

Following the observation of Herminghaus^21^, experimental and theoretical studies were conducted to investigate the underwater longevity of the plastron. Bobji *et al*.^25^ used total internal reflection (TIR) of light at the water-air interface to visualize the plastron and observed finite lifetimes for plastrons formed over regular and random surface patterns in quiescent condition. Poetes *et al*.^20^ used the same technique and reported that the lifetime of a thermodynamically instable plastron depends exponentially on the immersion height (hydrostatic pressure) and is comprised of two periods: (a) thinning of a flat plastron primarily due to diffusion of air into water, and (b) plastron break up into spherical cap bubbles. Jones *et al*. performed molecular dynamics simulations^22^, which along with the above mentioned TIR experiments, confirm the theoretical prediction of Lv *et al*.^23^ and Xu *et al*.^27^ that submicron roughness is essential to inhibit the condensation of water vapor phase inside the plastron and maintain a dry surface. Most recently, Lee *et al*.^7^ utilized TIR technique and obtained similar results to Poetes *et al*.’s^20^ in terms of plastron behavior under different immersion heights. Although remarkable progress has been made in characterizing the plastron behaviour, these investigations were carried out in quiescent condition, which casts doubt on their applicability in flowing fluids for realistic applications.

Most superhydrophobic surfaces with random texture exhibit virtually complete air coverage under quiescent water^5^,^20^,^28^, but once exposed to shear flow, the surface coverage plummets dramatically^28^. The liquid flow induces significantly higher air dissolution rates by altering the dominant mass transfer mechanism from diffusion to forced convection, which necessitates the investigation of plastron lifetime under shear flow. All experimental studies using light scattering technique^29^,^30^, direct visualization^29^,^31^ or quartz crystal microresonator^32^ confirm that the convection-diffusion mass transfer regime deteriorates the underwater longevity of the plastron and that this effect is intensified by increasing the flow rate. By applying a modified Blasius solution and integral method to the hydrodynamics and mass transfer equations, the theoretical study of Barth *et al*.^33^ quantitatively characterized the air loss for surfaces with regular patterns with varying gas fraction, respectively. Their study aim at examining the practicality of superhydrophobic surfaces subjected to one of the many environmental factors (i.e., shear flow) that reduce the plastron life-time.

One environmental factor that can be detrimental to the underwater longevity of plastron, is the wall collision of the particulate phase present in fresh and sea-water. Despite its critical importance, the effect of particle collision on the plastron lifetime has not been investigated. The particles, found in seawater, include less frequent particulates such as dirty bubbles (usually <100 μm)^34^, oil microdroplets^35^ and marine snow^36^, as well as microorganisms such as zooplanktons (ranging from 1 μm to a few mm) and phytoplanktons (ranging from 500 nm to 500 microns), with microgels and transparent exopolymer particles (TEP) at the other end of the spectrum^37^. TEPs are present in both fresh water and seawater and augment aggregation of solid micro-particles^38^. In fresh water environments (lakes and rivers) as well as water transport pipelines, the suspended particles mostly consist of clay (*d* < 4 μm), silt (4 < *d* < 62 μm) and fine sand (62 < *d* < 125 μm) particles. Particles with diameters greater than the roughness size or the entrapped bubbles, (e.g. dirty bubbles, large TEP and zooplanktons, etc.) can cause severe damages (to both plastron and the surface itself). Whereas due to lower inertia, microparticles are unlikely to cause any significant damage to the solid surface, and only the plastron is likely to be affected. Although they cause comparably worse damage, the large particles are much less common in nature compared to microparticles. Hence, the impact of these large particles should be differentiated from the impact of particles, which are as small as the roughness elements. Not only are micro-particles more abundant in nature, they are also used as tracers in PIV measurements investigating the drag reducing capability of the superhydrophobic surfaces (Ling *et al*.^26^ used 2 μm particles same as this work). Hence, these studies should consider the tracer-particle-induced deterioration of the superhydrophobicity in time.

In this work, we investigate the effect of viscous shear and suspending particulate phase by studying the underlying mechanisms responsible for the depletion of the air interlayer of a superhydrophobic surface subjected to a simplified underwater condition. An *in situ* non-invasive optical technique is developed, based on Samaha *et al*.’s^30^, to observe the longevity of the air layer over a superhydrophobic surface utilizing TIR at air-water interface. The lifetime of the plastron immersed in still water is obtained to provide information on the plastron shape and serves as a reference for comparison with the behavior in shear flow and particulate conditions. The shear flow is characterized by PIV and its influence on the lifetime of the plastron is investigated for under-saturated and over-saturated water (in terms of dissolved air content). To the authors’ knowledge, for the first time, experiments are conducted to study the influence of the wall impact of suspended micro-particles on the underwater longevity of the plastron. The mass transfer mechanisms are discussed and comparison of the experimental results with classical theory is presented.

## Results

### *In situ* non-invasive monitoring of the plastron kinetics

**Figure 1:**
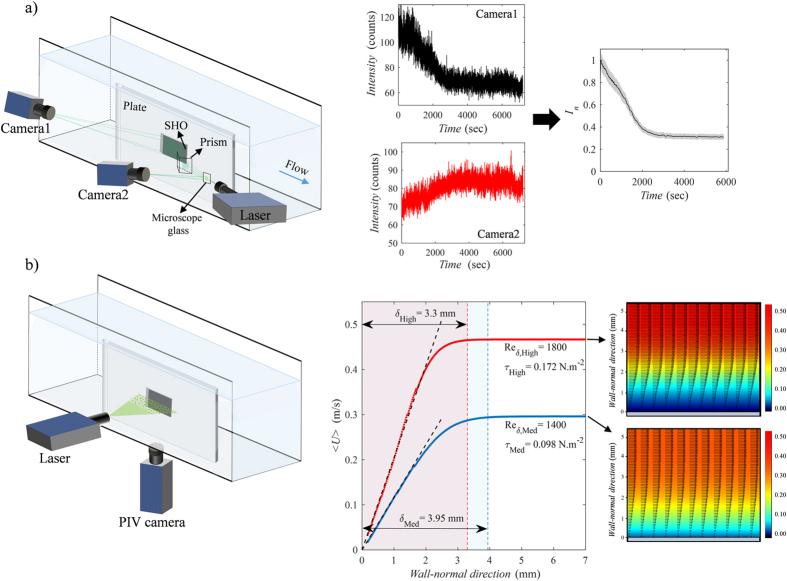
Schematic illustration of the experimental setups. (**a**) TIR experiment. The reflected light from the superhydrophobic surface is recorded by camera 1. The average intensity (each data point) corresponding to each image is calculated by averaging the image along vertical and horizontal direction leading to a single intensity value. The average intensity profile is then normalized by the average reference light intensity recorded by camera 2 from the beam reflected off the microscope glass slide. The normalized signals from first and second cameras are used to obtain the final intensity profile. (**b**) High-resolution PIV measurement of the laminar boundary layer. Linear fitting in the near-wall region is done to find the wall shear stress. In the vector fields, the color bars indicate the velocity magnitude.

The longevity of the air interlayer is analyzed using an optical apparatus developed based on TIR (details are discussed in the Methods section). The experimental apparatus is schematically illustrated in Fig. 1a and is based on the reflection of incident light from water/air interface when the incident angle is greater than the critical angle of total reflection. As has been reported in the literature, this phenomenon causes a silvery, mirror-like interface over the superhydrophobic surface^7^,^20^,^22^. The intensity of the reflected light from the plastron is measured by a CCD camera and normalized by a reference beam intensity recorded by a second camera to eliminate the fluctuations of the laser power as shown in Fig. 1b (detailed in Methods section). TIR only occurs at the water/air interface (plastron) and does not occur at the wetted spots of the surface. Hence, as the plastron decays, the intensity of the reflected light diminishes. This attenuation continues until complete transition from non-wetted (Cassie-Baxter) state to wetted (Wenzel) state takes place and an asymptotic state is reached. Timescales are extracted from the plot of intensity versus time to characterize the lifetime of the plastron and the mass transfer phenomena. Figure 1b illustrates the PIV setup used to characterize the boundary layer flow.

### Longevity of the superhydrophobic plastron immersed in quiescent water

**Figure 2:**
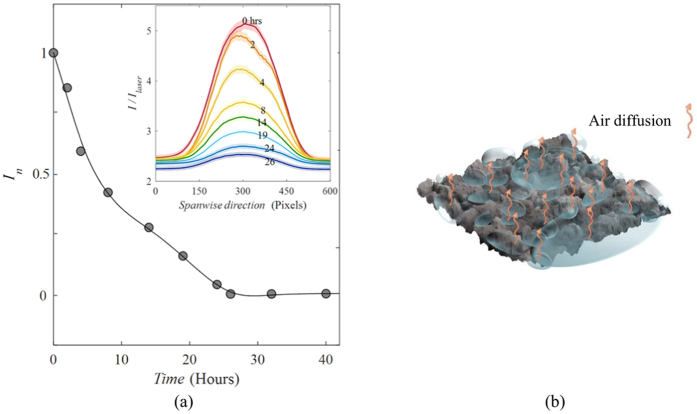
(**a**) Long-time behavior of plastron immersed in still water. The inset represents the variation of spanwise averaged intensity profiles in time (each pixel is 50 μm). (**b**) Schematic shape of the plastron consisting of spherical cap bubbles and larger planar regions. Vectors indicate the diffusion-dominated air dissolution into the still water.

The longevity of the plastron in underwater conditions has been investigated for a superhydrophobic sample immersed in stationary water. Other than delineating the air loss kinetics, which is useful for experimental and theoretical analysis of the mass transfer phenomena (details in Discussion), the time scale obtained from this experiment is used to infer the shape and size of the plastron through comparison with theory. Due to the slow rate of diffusion, the duration of air mass transfer from plastron to water was an order of magnitude longer than that of the forced convection cases (with flow). Hence, we collected ten sets of images (each set has 500 images) of the plastron decay during 40 hours. The average intensity of each set is calculated and presented compared to the immersion time in Fig. 2a. According to the figure, the plastron coverage starts to attenuate gently after immersion and continues until *t* = 26 hours, where the data reaches a plateau due to wetting. This behavior is in qualitative agreement with Bobji *et al*. and Samaha *et al*.’s observations although the lifetimes of their samples, 35 minutes and 75 hours, respectively, are different due to the different structures and chemistries of the surfaces. Recently, a different behavior has been reported^7^,^20^, which consists of an initial long plateau in intensity-time profile followed by a relatively fast decay of the plastron. Poetes *et al*.^20^ proved that this initial plateau is due to the thinning of a continuous planar plastron (Laplace pressure is zero) and that changes in rate of mass transfer is only dictated by hydrostatic pressure. The subsequent rapid decay of the plastron is attributed to the break up of the plastron into bubbles pinned to the surface during which Laplace pressure appears and dominates the plastron lifetime. According to the graph trend in Fig. 2a, the early start of the reduction in intensity suggests that the plastron mostly consists of spherical cap bubbles pinned to the surface. However, the 26-hour decay time of the plastron (compared to the observations of Poetes *et al*.^20^ and Lee *et al*.^7^) implies the coexistence of these bubbles with planar plastrons in a few regions on the surface (as illustrated schematically in Fig. 2b). Assuming the dominance of spherical cap bubbles, and based on Epstein and Plesset^39^, Ljunggren and Eriksson^40^ and Poetes *et al*.^20^, we adopted and modified an analogy between diffusion of air from the spherical caps and dissolution of air bubbles into water. Using the ideal gas law, the lifetime of spherical caps is obtained as


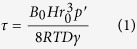


where *B*_*0*_ is the fractional volume of the spherical cap with respect to a sphere (~0.1), *H* is Henry’s constant, *r*_*0*_ is the initial bubble radius, *p’* is the equilibrium pressure (101.3 kPa), *R* is the gas constant, *T* is the temperature (294 K), *D* is the diffusion coefficient of air in water, and *γ* is the water surface tension. Using this equation and substituting the plastron lifetime value (26 hours), obtained from the experiments, the average bubble radius is estimated *r*_*0*_≈ 50 *μ*m.

### Shear flow-induced changes in longevity of the plastron

Most previous studies of plastron longevity under quiescent water have failed to predict the performance of the superhydrophobic surfaces under shear flow condition. This is mainly due to the change of mass transfer mechanism from diffusion to forced convection regime induced by the water flow. To characterize the convective mass transfer based on the universal characteristics of a canonical boundary layer flow, we have carried out high-magnification particle image velocimetry (PIV) measurements (schematically illustrated in Fig. 1b). Due to the high resolution, we were able to obtain accurate values of wall shear stress, boundary layer thickness (*δ*) and Reynolds number based on boundary layer thickness (Re_*δ*_) for the two considered flow rates. The values shown in the Fig. 1b represent a laminar boundary layer flow at the measurement location and by increasing the flow velocity, the shear stress increases by 75%.

**Figure 3:**
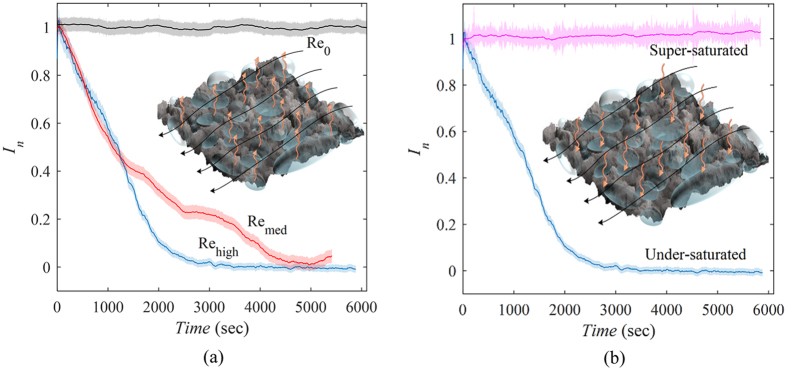
Normalized reflected light intensity profiles versus immersion time inside the flowing water channel. (**a**) Under-saturated state (95.0%), (**b**) over-saturated state (102.3%). In the insets, the black lines show the flow direction and the orange vectors show the direction of air transfer from/into plastron into/from flowing water. The thin data line indicates the moving average value and the shaded area illustrates the fluctuations.

Figure 3a demonstrates that once the superhydrophobic surfaces are exposed to flow (Re_med_ and Re_high_), the intensity of the reflected light from the surface attenuates gradually while the still water experiment (Re_0_) displayed no significant change during the 1.5-hour period. This indicates that the dominant mechanism of mass transfer has changed from free convection to forced convection, causing faster loss of the air interlayer and one order of magnitude shorter lifetime of the plastron compared to quiescent water, as shown schematically in the inset of Fig. 3a. This can also be observed in the ~50% reduction of the longevity of the superhydrophobicity for the higher Reynolds number (Re_high_) suggesting that, similar to the classical problems, higher velocity intensifies the forced mass convection. The observed behavior of the air interlayer subjected to canonical boundary layer flow is in qualitative agreement with the results of Samaha *et al*.^30^ who exposed the superhydrophobic samples to a jet flow. Each experiment was repeated three times and the results are presented in Table S1 in the Supplementary information. During these experiments, the dissolved air concentration was monitored and the air solubility of the water state was kept under-saturated with ~95.0% of the air concentration required for saturation at 19 °C (dissolved oxygen concentration was measured by YSI Model 52 DO meter, as detailed in Supplementary information).

### Shear flow-induced changes in longevity of the plastron inside super-saturated water

When the superhydrophobic sample is immersed in under-saturated water, the air interlayer dissolves into the water, causing a pressure drop inside the non-wetted surface pores and consequent water invasion. However, if the liquid is saturated, a phase equilibrium occurs between the plastron and the dissolved air in the ambient liquid and the plastron is sustained indefinitely^22^,^41^. To elucidate the behavior of superhydrophobicity in super-saturated ambient liquid, we conducted experiments inside flowing water containing ~2.3% air more than saturated state. This state was also identifiable through observation of small bubbles formed on the channel walls and the test plate. Figure 3b presents the variations of reflected light intensity subjected to water flow (Re_high_) for under-saturated and super-saturated ambient liquid. Although we expected an increase in reflected light intensity due to reverse mass transfer from dissolved air into the plastron (as illustrated in the inset of Fig. 3b), the super-saturated case here shows behavior similar to a saturated case since the air coverage remains constant. This behavior initially seems to be different from Dilip *et al*.’s observation where the entrapped air bubbles grew in size due to the reverse mass transfer^29^. This discrepancy is attributed to the vertical orientation of the plate and porous structure of our superhydrophobic surface. Due to the buoyancy force, the surface coverage and thickness of the plastron experiences a gradient in the vertical direction. The absorbed air from the ambient water into the plastron was pushed upwards forming a thick air layer on top of the sample. The accumulated excess air detaches from the surface as bubbles by shear-flow-induced pinch-off (extensive details in the Supplementary information). Consequently, the thickness of the plastron and the surface coverage is constant in time.

### Effect of particle-plastron collision on the longevity of the plastron

The presence and motion of suspended particles in water can influence the underwater longevity of the plastron. This effect stems from the shortcoming of the particles in following the streamlines in the vicinity of the wall. The streamlines of the flow field in the vicinity of the wall are expected to be slightly curved due to the roughness elements. Therefore, larger and/or faster particles with longer relaxation time (longer time response to acceleration/deceleration of the surrounding fluid), develop a slip velocity relative to the fluid motion and deviate from the curved flow streamlines and collide with the surface^42^.

**Figure 4:**
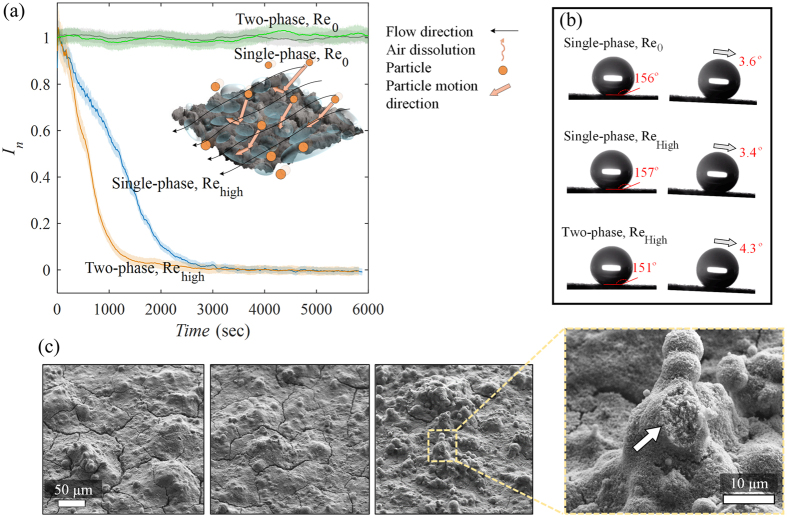
Effect of addition of particles on lifetime, wetting, and physical properties of the superhydrophobic surface. (**a**) Normalized reflected light intensity profiles versus immersion time of the superhydrophobic surface inside flowing water channel for single and two-phase systems. In the schematic of the inset, the black lines show the flow direction and orange circles represent the immersed particles. The direction of air transfer is similar to the inset of Fig. 3a. (**b**) Wetting properties of the samples subjected to no-flow (Re_0_), single-phase, and two-phase flows after drying. Contact angle and roll-off angle of the samples were measured. The samples subjected to two-phase flow displayed lower contact-angle and higher roll-off angle implying possible damage to the surface. (**c**) SEM images of the samples subjected to no-flow, single-phase and two-phase flows, respectively, from left to right. The zoomed photo displays the damage on the surface subjected to two-phase flow due to particle impact.

Due to prevalence of micro-scale particles in nature, we selected 2 μm (average size) silver-coated glass spheres (density: 4 gr/cm^3^) with a concentration of 20–30 particles per mm^−3^ quantified through tomographic PIV reconstruction^43^. A dilute mixture was formed in which particle motion is dictated by the fluid flow but not vice versa (i.e., one-way coupling)^42^. This condition conforms to most of the practical applications in the environment (fresh and sea water) as well as to the tracer density required for particle image velocimetry. Despite the rather small size, these particles are prone to deviate during curvilinear motions due to their high density. The larger particles (>5 μm), of which there are fewer in the distribution, have a Stokes number around 1 and deviate from most of the flow streamlines in sudden deceleration incidents. Figure 4a presents the reflected light intensity profiles of the identical samples subjected to single-phase (without particles) and two-phase (with particles) flows at Re_0_ and Re_high_. Initially, the Re_0_ case was measured to elucidate the probable physicochemical effects of the addition of particles to the thermodynamics of the plastron. As shown in the figure, the addition of particles does not change the behavior of the air interlayer inside quiescent water. In contrast, it is apparent that the lifetime of the plastron has declined by 50% inside the two-phase system with Re_high_ compared to the single-phase system, indicating the significant effect of motion of suspended particles on plastron longevity. The inertia of this particle impact induces disturbances, which could destabilize the bubbles attached to the solid surface causing a recession of wetting lines and a subsequent invasion of water into the pores. Measurements of contact angle and roll-off angle were also carried out on the superhydrophobic surfaces after the light reflection experiments (surfaces being wet and dried again). As shown in Fig. 4b, the samples exhibit similar wetting behavior irrespective of the shear flow magnitude in the single-phase cases. On the other hand, the sample exposed to two-phase flow, displayed smaller contact angle. Scanning electron microscopy (SEM) was conducted to examine any possible surface changes/damages (Fig. 4c). Unlike the samples immersed in a single-phase system, the sample in the two-phase system experienced sporadic damage on highest roughness elements. These elements are more susceptible to particle impact due to their height since they protrude through the air-water interface immediately after immersion. This type of damage is not observed for roughness elements with lower heights.

## Discussion

We monitored the dissolution kinetics of air plastron subjected to quasi-realistic underwater conditions to examine the effect of shear stress and particulate phase on the longevity of the plastron. The significant influence of shear flow on the lifetime of the plastron implied a possible change in the mass transfer regime. Furthermore, the proof-of-concept experiments to delineate the effect of suspending micro-particles revealed a noticeably detrimental impact on the underwater longevity of the plastron. The random structure of the superhydrophobic surface makes the derivation of a direct theoretical analysis for forced convection challenging. Hence, to provide insight into the plastron shape and serve as a reference for analytical analysis of flow-induced mass transfer mechanisms, we measured the plastron lifetime immersed in still water (natural convection). To this end, we have adopted a modified version of Samaha *et al*.’s^30^ argument on convective mass transfer from plastron. For the case of a superhydrophobic surface immersed in quiescent water, the air dissolves into water through natural convection mass transfer driven by the wall-normal gradient of dissolved air concentration. Due to low solubility of air in water, Henry’s law can be used (details in Samaha *et al*.^30^). The Grashof number,


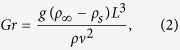


indicates the ratio of buoyancy forces to viscous forces. *ρ*_*∞*_and *ρ*_*s*_denote the density of water away from the surface and at the surface, respectively (variation is due to the dissolved air). *L* is the characteristic length scale (here length of the measurement region). *ρ* is the aggregate density of air and water mixture and *ν* is the water kinetic viscosity. The Schmidt number, *Sc*, represents the ratio of momentum diffusivity to molecular diffusion rate and is calculated from


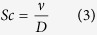


where *D* is the mass diffusion coefficient of air in water. Based on these two numbers, the Rayleigh number is determined as


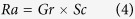


and subsequently, the Sherwood number, *Sh*, which indicates the ratio of the total rate of mass transfer to the rate of molecular diffusive mass transfer, is found from this correlation for natural convection from a vertical plate^44^


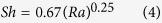


According to Samaha *et al*.^30^, the total mass of air dissolved into water (through either natural or forced convection), *m*_*a*_, is


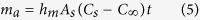


**Figure 5:**
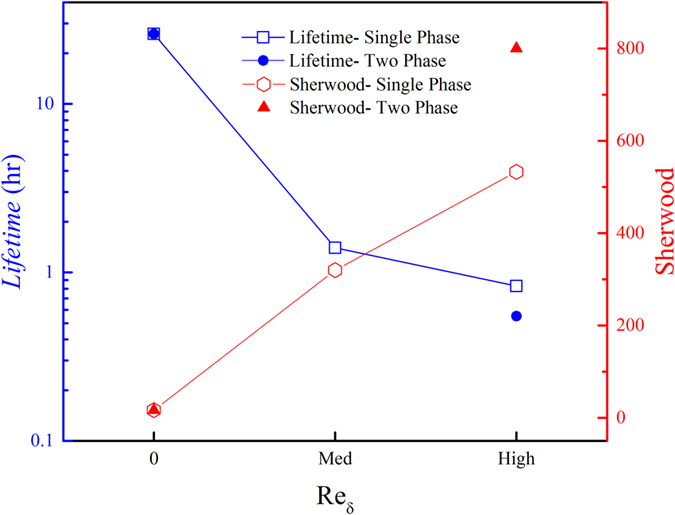
Lifetime and Sherwood number comparison for samples subjected to single-phase and two-phase flows.

*m*_*a*_ is relatively cons*t*an*t* for all identical superhydrophobic surfaces immersed in water with the same dissolved air content. Here, *h*_*m*_ is the mass transfer convection coefficient, *A*_*s*_ is the sample surface area and (C_*s*_−C_∞_) and *t* represent dissolved air concentration and immersion time, respectively. Assuming that the surface area and the concentration gradient are the same for all cases, we can reason that the forced convection only alters the convective mass transfer coefficient. Hence,


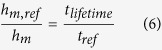


where *ref* denotes the natural convection (still water) case. Knowing that


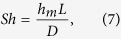


using equations 2–6 and by finding the *t*_*ref*_ and *t*_*lifetime*_ from the experiments, we can find the *Sh* number for forced convection cases. The results allow a quantitative comparative study of the effects of the quasi-environmental factors in mass transport from the plastron. The lifetimes obtained from the experimental data and Sherwood numbers, estimated by theoretical analysis, are presented compared to the Reynolds number in Fig. 5. Compared to natural convection (Sh = 17), the Sherwood number has increased by 20 and 30 times for Re_δ,med_ = 1400 and Re_δ,high_ = 1800, respectively. The results macroscopically indicate that the change in the mass transfer regime from natural to forced convection drastically increases the air dissolution rate in a laminar boundary layer flow. This effect is expected to intensify for turbulent flow where turbulent motions enhance mixing and increase wall friction. Furthermore, the ~50% increase in mass transfer rate for two-phase flow (particles suspending in water) compared to single-phase proves the concept that presence and motion of micro-scale particles inside water can significantly decrease the lifetime of the air plastron by enhancing air mass transfer from plastron. Next to shear flow, the particle-plastron collision imposes another constraint to practicality of current superhydrophobic surfaces in real-life applications such as drag reduction in marine vessels and pipelines.

In conclusion, we have conducted *in-situ* monitoring of the air loss kinetics from a plastron subjected to single- and two-phase flows, which are similar to practical applications in fresh and sea water environments. The canonical laminar boundary layer shortens the lifetime of the plastron due to the change of mass transfer regime from diffusion dominated regime to forced convection and the effect increases with Reynolds number. The critical role of the concentration of dissolved air was proven by the lack of any response in a similar flow condition inside super-saturated water. The experiments in the two-phase flow demonstrate that the flow-induced particle-plastron collision reduces the lifetime of the plastron by ~50%. Theoretical analysis based on the plastron behavior in quiescent environment provides a quantitative comparison between the underlying mass transfer mechanisms. From our results, we suggest more efforts towards design of superhydrophobic surfaces with sustainable air supply (e.g., air restoration systems^26^), higher mechanical strength, and optimal roughness structure for better entrapment of air pockets. The latter can be achieved through mimicking the Salvinia effect observed in the floating ferns of genus *Salvinia*. In addition to the ubiquitous hierarchical structure, this plant has two additional remarkable properties: first, elasticity in hair-like structures which allows the compression of the plastron in response to dynamic pressure fluctuations^45^ and second, a paradoxical chemical heterogeneity (Salvinia paradox) consisting of hydrophilic hair tips combined with the superhydrophobic base^46^,^47^. This effect causes the pinning of the air-water interface to the hydrophilic tips leading to longer lifetime of the plastron.

## Methods

### Experimental apparatus and procedure for flow characterization

A laminar boundary layer is formed over the surface of a two-dimensional hydrofoil with an elliptical leading edge (aspect ratio of 5:1) installed vertically inside a water channel. The superhydrophobic surface is placed as a replaceable module with 50 mm × 50 mm on the hydrofoil surface 30 cm downstream of the leading edge and at a depth of 15 cm under the free surface of the water channel. The dimension of the superhydrophobic module is large enough to separate the measurement region from any edge effect observed within a few millimeters from the sample boarder. The experiments are carried out at two different flow rates with free stream velocities of *U*_*∞*_ = 0.30 m/s and 0.47 m/s corresponding to Re_δ_ = 1400 and 1800 (based on the local boundary layer thickness), respectively. The selected parameters (downstream distance and velocity) ensure presence of a laminar boundary layer as characterized using planar PIV measurement. The illumination was carried out by a dual-cavity Nd-YAG laser (Solo III-15 New Wave Research) with maximum output of 200 mJ/pulse and a wavelength of 532 nm. The water channel was seeded with 2 μm silver-coated glass spheres (Potters Industries Conduct-O-Fil® SG02S40). A CCD camera with a sensor size of 1376 × 1040 pixels and 12 bit resolution (Imager Intense, LaVision) was applied to capture the images. The camera is equipped with a 105 mm objective with an aperture setting of *f*/5.6. For each data set 5000 double-frame images were recorded with Δ*t* = 200 μs. The camera recorded a 10 mm × 10 mm field-of-view with digital resolution of 137 pixel mm^−1^. The PIV images were improved for the cross-correlation algorithm by subtracting the ensemble minimum from the individual images. The images were further normalized by the ensemble average of the recordings. The mean velocity profile was obtained by averaging the cross-correlations over the ensemble of recordings^48^. This method increases the signal-to-noise ratio which allows a higher spatial resolution by allowing a smaller Gaussian interrogation window (IW) of 12 × 12 pixels with 75% overlap elongated with 4:1 aspect ratio in the streamwise direction leading to 372 × 372 vectors per field.

### Experimental apparatus and procedure for *in situ* monitoring of the plastron

The optical apparatus to monitor the kinetics of the plastron was developed based on Samaha *et al*.’s experimental setup^30^. However, it was improved to eliminate the fluctuations and/or alteration of the light source power. The laser beam with circular cross-section of 3 mm in diameter was generated by New Wave Solo I-15 Hz laser at an angle of 50° with respect to the sample superhydrophobic surface. The beam passes through a microscope glass which reflects about 1% of the laser intensity as the reference beam toward Camera 2 to record the fluctuations and variations of the laser power as shown in Fig. 1a. The laser beam is directed into two cylindrical lenses to expand to a rectangular cross-section of 5 cm × 4 cm and illuminate the full height of the superhydrophobic surface. The partially expanded beam was passed through a prism with perpendicular walls into the water channel. After being reflected from the plastron, its intensity was measured by Camera 1 as shown in Fig. 1a. The frequency of the laser and cameras was set to 0.1 Hz.

### Image analysis and signal processing

A script has been developed in MATLAB (the Mathworks) to analyze the images collected by the two cameras. The intensity of images of the light reflected from the plastron was averaged in horizontal and vertical directions resulting in average reflection intensity, *I*_avg_. The same procedure was applied to the images collected by the second camera associated with the reference beam (*I*_*laser, avg*_). The normalized average intensity was obtained as


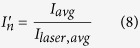


which, eliminates the inconsistencies attributed to the variations of laser power. The plateau value (associated with fully wet state), *I*′_*n,plateau*_, was subtracted from the normalized average intensity and divided by the maximum value of the image ensemble to set the range of changes for all intensity profiles from zero to one.


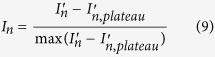


This normalized intensity value was plotted as the intensity profile of the individual superhydrophobic samples in Figs 3 and 4a. Additionally, a moving average with a kernel of 10 was applied to remove high frequency fluctuations. The shaded boundaries indicate the range of fluctuations and the moving average is depicted as a solid line inside the shade area. The lifetime of the plastron is estimated as the time duration for the average normalized intensity (*I*_n_) to reach zero.

### Superhydrophobic surface fabrication and characterization

**Figure 6:**
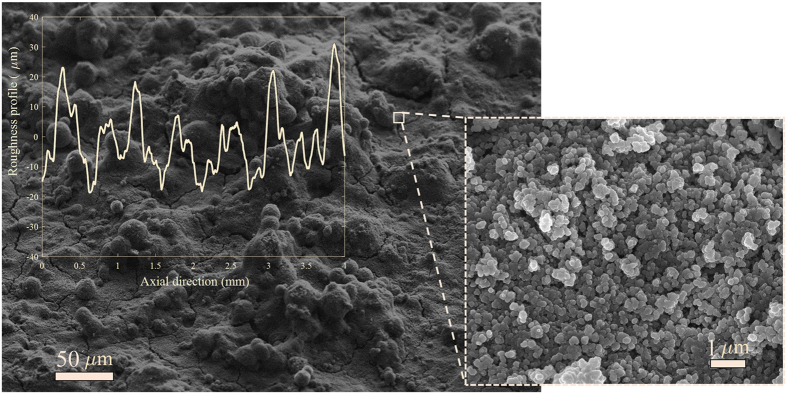
SEM image of the superhydrophobic surface with 200× and 10,000× magnifications. The microscale roughness of the surface measured by the profilometer is also added.

The random superhydrophobic surfaces were fabricated via spray-coating. The commercially available superhydrophobic coating, NeverWet®, was selected due to its proven capability to modify turbulent flow structures and consequently reduce drag^11^. MSDS and energy dispersive spectroscopy (EDS) analysis show that the coating consists of a silicone-based under-coat layer containing microparticles and a surface coating of hydrophobic silica nanoparticles. The wet surfaces were stored in the laboratory environment for a few days to dry before the contact angle measurement and the SEM imaging. The SEM images of the surface with two different magnifications are presented in Fig. 6. The micro-scale roughness profile of the system was measured by Ambios XP-300 surface profilometer with a resolution of 0.1 μm. The roughness profile is also added to Fig. 6. Statistical analysis of the roughness measurement yielded 7.9, 10.2 and 41.0 μm for arithmetic surface roughness, average root-mean-square height and mean peak-to-trough roughness height, respectively (details of calculation presented in Supplementary information). For detailed description of the fabrication and characterization process refer to Hokmabad *et al*.^11^.

## Additional Information

**How to cite this article**: Hokmabad, B. V. and Ghaemi, S. Effect of Flow and Particle-Plastron Collision on the Longevity of Superhydrophobicity. *Sci. Rep.* **7**, 41448; doi: 10.1038/srep41448 (2017).

**Publisher's note:** Springer Nature remains neutral with regard to jurisdictional claims in published maps and institutional affiliations.

## Supplementary Material

Supplementary Information

## References

[b1] Lauga E. & Stone H. A. Effective slip in pressure-driven Stokes flow. J. Fluid Mech. 489, 55–77 (2003).

[b2] Marmur A. Underwater superhydrophobicity: Theoretical feasibility. Langmuir 22, 1400–1402 (2006). 16460052 10.1021/la052802j

[b3] Bocquet L. & Lauga E. A smooth future? Nat. Mater. 10, 334–337 (2011). 21505463 10.1038/nmat2994

[b4] Lafuma A. & Quéré D. Superhydrophobic states. Nat. Mater. 2, 457–60 (2003). 12819775 10.1038/nmat924

[b5] Srinivasan S. . Sustainable drag reduction in turbulent Taylor-Couette flows by depositing sprayable superhydrophobic surfaces. Phys. Rev. Lett. 114, 1–5 (2015). 10.1103/PhysRevLett.114.014501 25615472

[b6] Brennan J. C. . Flexible conformable hydrophobized surfaces for turbulent flow drag reduction. Sci. Rep. 5, 10267 (2015). 25975704 10.1038/srep10267 PMC4432562

[b7] Lee B. J. . Bio-inspired dewetted surfaces based on SiC/Si interlocked structures for enhanced-underwater stability and regenerative-drag reduction capability. Sci. Rep. 6, 24653 (2016). 27095674 10.1038/srep24653 PMC4837397

[b8] Lee C., Choi C. H. & Kim C. J. Structured surfaces for a giant liquid slip. Phys. Rev. Lett. 101, 1–4 (2008). 10.1103/PhysRevLett.101.064501 18764458

[b9] Teisala H., Tuominen M. & Kuusipalo J. Superhydrophobic Coatings on Cellulose-Based Materials: Fabrication, Properties, and Applications. Adv. Mater. Interfaces 1, 1–20 (2014).

[b10] Liu T. & Kim C. J. Turning a surface superrepellent even to completely wetting liquids. Science 346, 1096–1100 (2014). 25430765 10.1126/science.1254787

[b11] Hokmabad B. V. & Ghaemi S. Turbulent flow over wetted and non-wetted superhydrophobic counterparts with random structure. Phys. Fluids 28, 15112 (2016).

[b12] Park H., Sun G. Y. & Kim C. J. Superhydrophobic turbulent drag reduction as a function of surface grating parameters. J. Fluid Mech. 747, 722–734 (2014).

[b13] Joseph P. . Slippage of water past superhydrophobic carbon nanotube forests in microchannels. Phys. Rev. Lett. 97, 1–4 (2006). 10.1103/PhysRevLett.97.156104 17155344

[b14] Rothstein J. P. Slip on Superhydrophobic Surfaces. Annu. Rev. Fluid Mech 42, 89–109 (2010).

[b15] Koch K. & Barthlott W. Superhydrophobic and superhydrophilic plant surfaces: an inspiration for biomimetic materials. Philos. Trans. A. Math. Phys. Eng. Sci. 367, 1487–509 (2009). 19324720 10.1098/rsta.2009.0022

[b16] Zhang P., Lin L., Zang D., Guo X. & Liu M. Designing Bioinspired Anti-Biofouling Surfaces based on a Superwettability Strategy. Small, doi: 10.1002/smll.201503334 (2016). 26917251

[b17] Bocquet L. & Barrat J.-L. Flow boundary conditions from nano-to micro-scales. Soft Matter 3, 685–693 (2007). 10.1039/b616490k 32900128

[b18] Ou J., Perot B. & Rothstein J. P. Laminar drag reduction in microchannels using ultrahydrophobic surfaces. Phys. Fluids 16 (2004).

[b19] Truesdell R., Mammoli A., Vorobieff P., Van Swol F. & Brinker C. J. Drag reduction on a patterned superhydrophobic surface. Phys. Rev. Lett. 97, 1–4 (2006). 10.1103/PhysRevLett.97.044504 16907578

[b20] Poetes R., Holtzmann K., Franze K. & Steiner U. Metastable underwater superhydrophobicity. Phys. Rev. Lett. 105, 1–4 (2010). 10.1103/PhysRevLett.105.166104 21230986

[b21] Herminghaus S. Roughness-induced non-wetting. Eur. Lett. 52, 165–170 (2000).

[b22] Jones P. R. . Sustaining dry surfaces under water. Sci. Rep. 5, 12311 (2015). 26282732 10.1038/srep12311 PMC4539549

[b23] Lv P., Xue Y., Shi Y., Lin H. & Duan H. Metastable states and wetting transition of submerged superhydrophobic structures. Phys. Rev. Lett. 112 (2014). 10.1103/PhysRevLett.112.196101 24877948

[b24] Lee C. & Kim C. J. Underwater restoration and retention of gases on superhydrophobic surfaces for drag reduction. Phys. Rev. Lett. 106, 1–4 (2011). 10.1103/PhysRevLett.106.014502 21231747

[b25] Bobji M. S., Kumar S. V., Asthana A. & Govardhan R. N. Underwater sustainability of the ‘Cassie’ state of wetting. Langmuir 25, 12120–12126 (2009). 19821621 10.1021/la902679c

[b26] Ling H. . High-resolution velocity measurement in the inner part of turbulent boundary layers over super-hydrophobic surfaces. J. Fluid Mech. 801, 670–703 (2016).

[b27] Xu M., Sun G. & Kim C. Infinite Lifetime of Underwater Superhydrophobic States. Phys. Rev. Lett. 113, 136103 (2014). 25302907 10.1103/PhysRevLett.113.136103

[b28] Moreira D., Park S., Lee S., Verma N. & Bandaru P. R. Dynamic superhydrophobic behavior in scalable random textured polymeric surfaces. J. Appl. Phys. 119, 125302 (2016).

[b29] Dilip D., Bobji M. S. & Govardhan R. N. Effect of absolute pressure on flow through a textured hydrophobic microchannel. Microfluid. Nanofluidics 19, 1409–1427 (2015).

[b30] Samaha M. A., Tafreshi H. V. & Gad-El-Hak M. Influence of flow on longevity of superhydrophobic coatings. Langmuir 28, 9759–9766 (2012). 22639940 10.1021/la301299e

[b31] Xiang Y., Xue Y., Lv P., Li D. & Duan H. Influence of fluid flow on the stability and wetting transition of submerged superhydrophobic surfaces. Soft Matter 12, 4241–4246 (2016). 27071538 10.1039/c6sm00302h

[b32] Lee M. . Highly stable superhydrophobic surfaces under flow conditions. Appl. Phys. Lett. 106, 11605 (2015).

[b33] Barth C. A., Samaha M. A. & Tafreshi H. V. Convective Mass Transfer From Submerged Superhydrophobic Surfaces. Int. J. Flow Control 5, 79–88 (2013).

[b34] Woolf D. K. Fluxes at the Sea Surface. IR Radiometers. Langmuir Circulation and Instability. Surface, Gravity and Capillary Waves. Wave Generation by Wind. Whitecaps and Foam. J. Phys. Oceanogr. 22, 412 (2001).

[b35] Feng J. . Nanoemulsions obtained via bubble-bursting at a compound interface. Nat. Phys. 10, 606–612 (2014).

[b36] Bochdansky A. B., Clouse M. A. & Herndl G. J. Dragon kings of the deep sea: marine particles deviate markedly from the common number-size spectrum. Sci. Rep. 6, 22633 (2016). 26940454 10.1038/srep22633 PMC4778057

[b37] Azam F. & Malfatti F. Microbial structuring of marine ecosystems. Nat. Rev. Microbiol. 5, 782–791 (2007). 17853906 10.1038/nrmicro1747

[b38] Meng S. & Liu Y. New insights into transparent exopolymer particles (TEP) formation from precursor materials at various Na+/Ca 2+ ratios. Sci. Rep. 6, 19747 (2016). 26790536 10.1038/srep19747 PMC4726276

[b39] Epstein P. S. & Plesset M. S. On the Stability of Gas Bubbles in Liquid-Gas Solutions. J. Chem. Phys. 18, 1505 (1950).

[b40] Ljunggren S. & Eriksson J. C. The lifetime of a colloid-sized gas bubble in water and the cause of the hydrophobic attraction. Colloids Surfaces A Physicochem. Eng. Asp. 129, 151–155 (1997).

[b41] Vakarelski I. U., Chan D. Y. C., Marston J. O. & Thoroddsen S. T. Dynamic air layer on textured superhydrophobic surfaces. Langmuir 29, 11074–11081 (2013). 23919719 10.1021/la402306c

[b42] Guha A. Transport and Deposition of Particles in Turbulent and Laminar Flow. Annu. Rev. Fluid Mech. 40, 311–341 (2008).

[b43] Hokmabad, B. V. Turbulent Flow over a Superhydrophobic Surface with Isotropic Slip. (University of Alberta). doi: Record ID oai:alberta:g445ch13x, 2015.

[b44] Zaki M., Nirdosh I. & Sedahmed G. Natural Convective Mass-Transfer Behavior of Horizontal and Vertical Perforated Surfaces. Ind. Eng. Chem. Res. 41, 3307–3311 (2002).

[b45] Ditsche P. . Elasticity of the hair cover in air-retaining Salvinia surfaces. Appl. Phys. A 121, 505–511 (2015).

[b46] Barthlott W. . The Salvinia Paradox: Superhydrophobic Surfaces with Hydrophilic Pins for Air Retention Under Water. Adv. Mater. 22, 2325–2328 (2010). 20432410 10.1002/adma.200904411

[b47] Amabili M., Giacomello A., Meloni S. & Casciola C. M. Unraveling the Salvinia Paradox: Design Principles for Submerged Superhydrophobicity. Adv. Mater. Interfaces 2, 1500248 (2015).

[b48] Meinhart C. D., Wereley S. T. & Santiago J. G. A. PIV Algorithm for Estimating Time-Averaged Velocity Fields. J. Fluids Eng. 122, 285–289 (2000).

