# Supplementary material for: Effect of Flow and Particle-Plastron Collision on the Longevity of Superhydrophobicity
Source: Sci Rep. 2017 Jan 27;7:41448. doi: 10.1038/srep41448 (PMC5269735; doi:10.1038/srep41448)
Supplement: Supplementary Information [file srep41448-s1.pdf]

Supplementary information:

Effect of Flow and Particle-Plastron Collision on the Longevity of Superhydrophobicity

Babak Vajdi Hokmabad, Sina Ghaemi

Lifetime of superhydrophobic surfaces: The mean values for three repeats of each measurement are presented in Table S1 along with the corresponding standard deviation.

Dissolved oxygen measurement: In order to find the state of the water in terms of air content (under-saturated/saturated/over-saturated) we measured the concentration of dissolved oxygen in the water as a representative of air content. The concentration of oxygen dissolved in the channel water was measured and monitored in real-time by YSI Model 52 DO meter. The measured values were compared to saturation values in literature and the under/over saturation percentages were obtained. The over-saturated state was obtained by running the flow loop for a few hours so the water temperature raised and air solubility decreases.

Plastron gradient and air accumulation: As discussed in the manuscript, due to the vertical orientation of the superhydrophobic surface and its porous structure, buoyancy pushes the air layer upwards and the surface coverage and thickness of the plastron experiences a gradient in the vertical direction. This is shown in Fig. S1 which shows intensity profiles averaged over streamwise direction for non-wetted and wetted states. The gradient in vertical direction is due to both increase in plastron thickness and coverage as well as the skewness of the laser profile. By comparing the two profiles, we can eliminate the effect of laser profile skewness. It is evident that the high gradient in the upper half of the plastron diminishes more than the lower half, implying that upper half contains more air and consequently, air loss is more noticeable there. The air, which is pushed upwards, causes accumulation of a thick air layer on top of the sample (Fig. S2) where this excessively accumulated air leaves the surface by shear- induced pinch-off of bubbles (Fig. S3). This is why, for the case of over-saturated flow the thickness of the plastron and the surface coverage is constant in time.

Surface roughness characterization: Top view SEM images of the superhydrophobic surfaces have been presented in Fig. S4 with different magnifications. Fig. S5 presents the SEM images taken from a 45° angle in order to observe the roughness elements on the surface.

The roughness numbers presented in the paper are calculated from the measurements of Ambios XP-300 surface profilometer with a resolution of 0.1 μm. The surface roughness R_a, arithmetic roughness, is calculated as

$$R_a = \frac{1}{L}\int_0^L \left| Z(x) - \overline{Z} \right| dx,$$

where $Z(x)$ is the height of the surface roughness elements, L is the sampling length, and $\overline{Z}$ is the mean of the measured profile $Z(x)$.

The average root-mean-square height, R_{rms}, of the profiles is calculated as

$$R_{rms} = \sqrt{\frac{1}{L}\int_0^L \left|Z(x) - \overline{Z}\right|^2 dx} \ .$$

The mean peak-to-trough roughness parameter is found by averaging the maximum peak-to-trough heights of the surface roughness within five successive sampling sections of the profiles measured:

$$R_{PT} = \frac{1}{5}\left(Z_1 + Z_2 + Z_3 + Z_4 + Z_5\right)$$

where Z_i is the maximum peak-to-trough height in *i*th sampling section.

Table S1. Lifetime of the samples (seconds) subjected to different flow conditions

	**Single-phase**	**Two-phase**
No flow	*Mean*: ~94,000<br>*St.Dev*: -	*Mean*: ~94,000<br>*St.Dev*: -
$\mathrm{Re}_{\mathrm{med}}$ = 1,400	*Mean*: 4,640<br>*St.Dev*: 320	-<br>-
$\mathrm{Re}_{\mathrm{high}}$ = 1,800	*Mean*: 2,540<br>*St.Dev*: 110	*Mean*: 1,570<br>*St.Dev*: 210

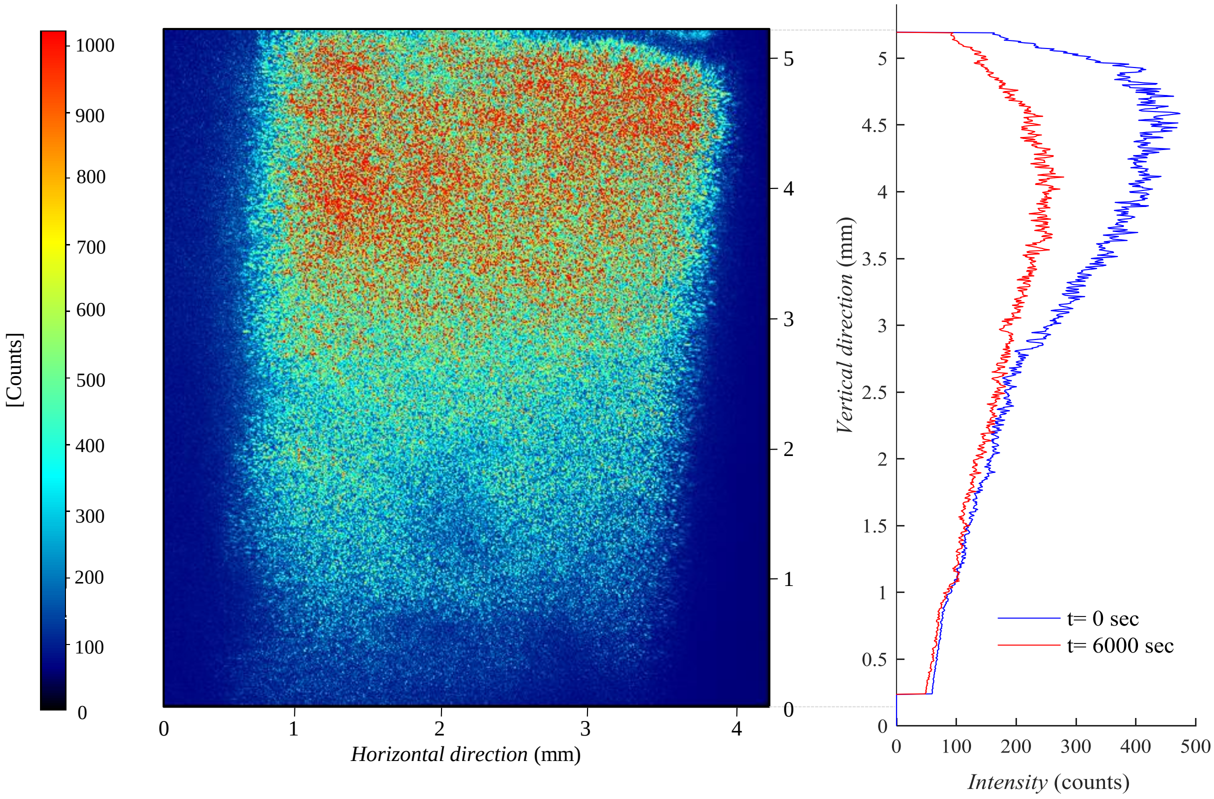

Figure S1. The light intensity reflected from the full height of the sample (left) and the intensity profiles averaged over horizontal direction (right). The profiles are presented for t = 0 sec in which flow has not affected the plastron and t = 6000 sec in which the surface is wet.

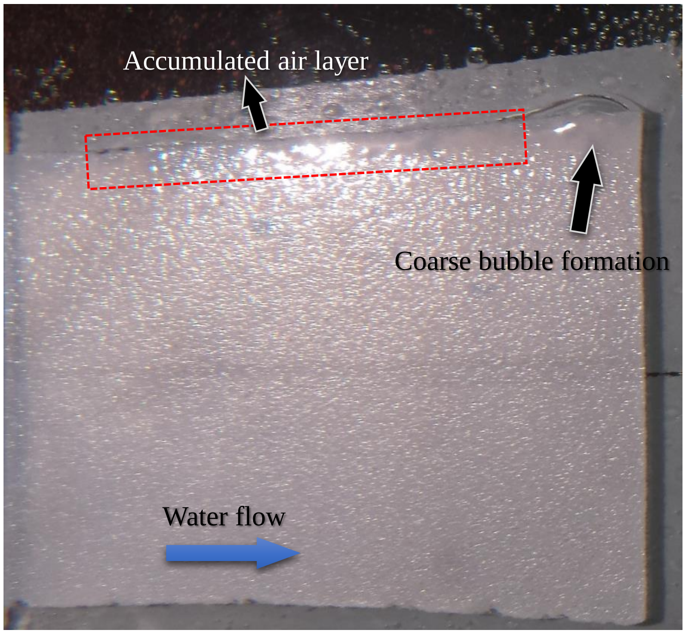

Figure S2. Air accumulation at the top of the samples

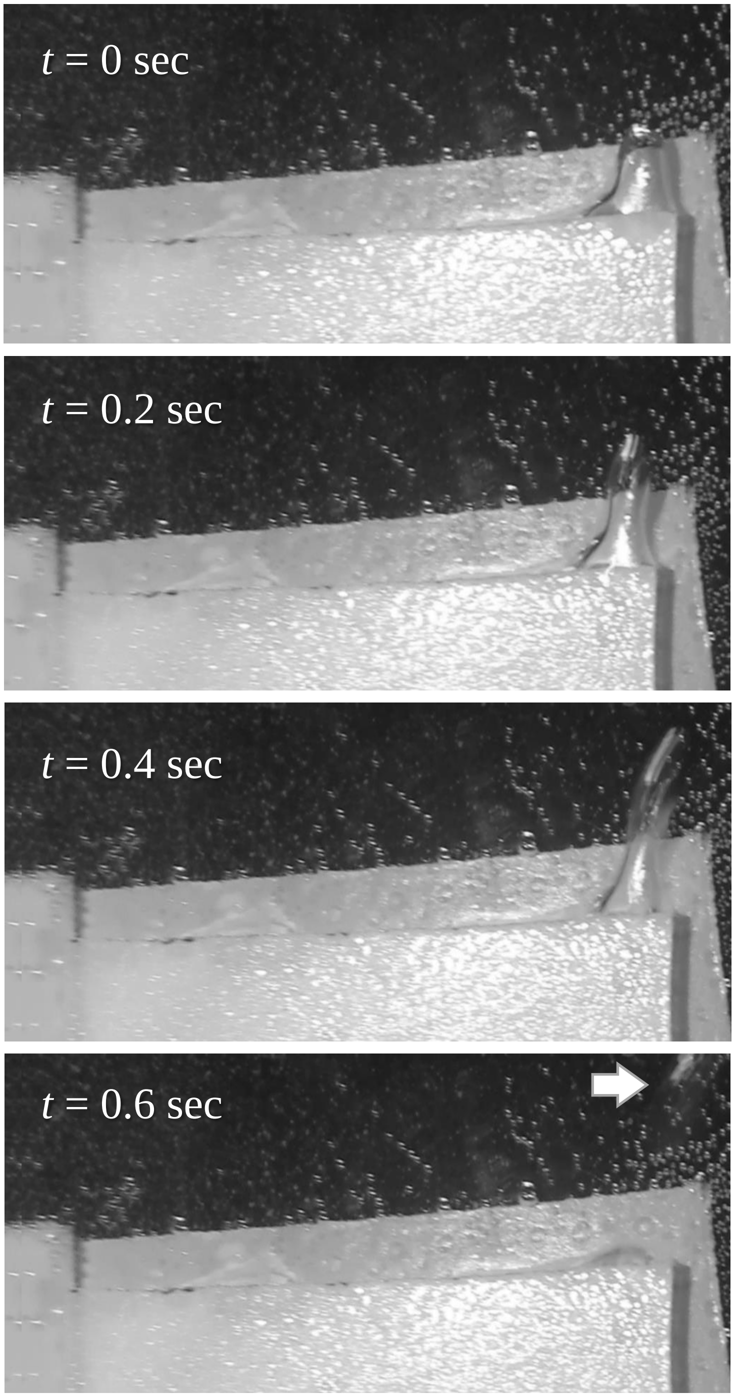

Figure S3. Air bubble formation and shear flow-induced pinch-off at top of the samples

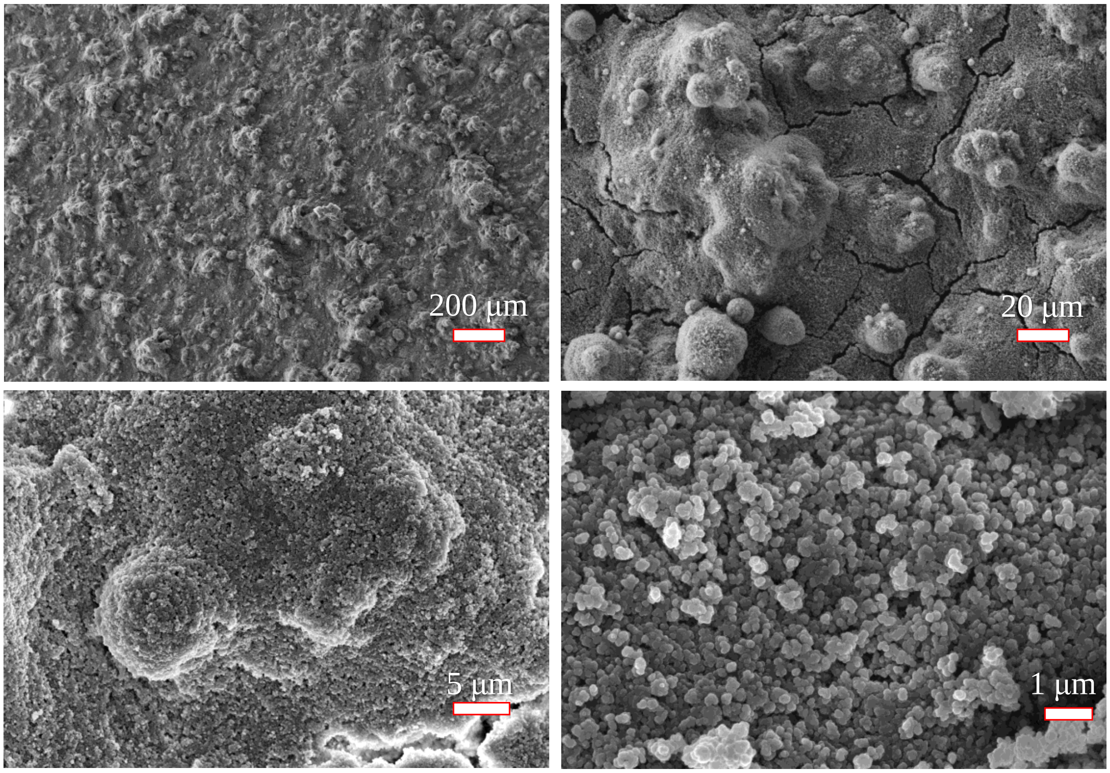

Figure S4. Top-view SEM images of the samples before exposing to flow. Both micro-scale roughness elements and the hydrophobic nanoparticles (the top coating) are shown.

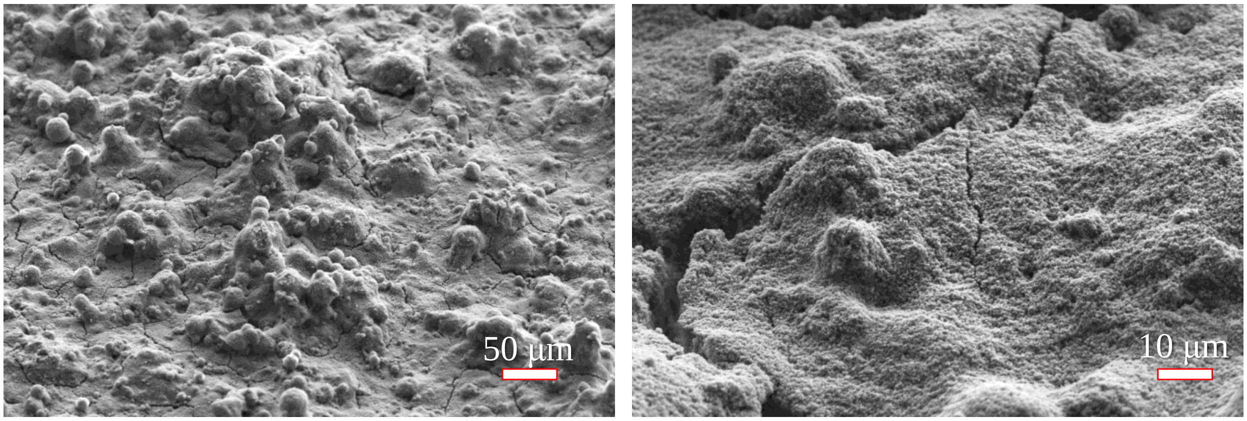

Figure S5. Tilted view of the samples before exposing to flow. A rough surface has formed with a full coverage by hydrophobic nanoparticles.
